# CAR-T细胞联合PD-1单抗治疗高危慢性淋巴细胞白血病合并Richter转化2例报告并文献复习

**DOI:** 10.3760/cma.j.issn.0253-2727.2023.05.013

**Published:** 2023-05

**Authors:** 进 周, 香萍 宗, 莹 张, 洪智 耿, 彩霞 李

**Affiliations:** 苏州大学附属第一医院血液科，苏州 215006 Department of Hematology, the First Affiliated Hospital of Soochow University, Suzhou 215006, China

慢性淋巴细胞白血病（CLL）是一种慢性淋巴细胞增殖性疾病，其异质性很高，不同患者预后差别很大。CLL国际预后指数（CLL-IPI）将CLL患者分为低危、中危、高危与极高危组，其中极高危组预后较差，5年生存率仅有23.3％。另外有10％～15％的CLL患者会发生Richter转化（RT）。一旦发展为RT，患者的中位生存期往往不超过1年。尽管既往免疫化疗及小分子靶向药物对部分CLL患者有效，但仍然难以克服具有高危遗传学特征患者的不良预后。本研究我们报道2例伴TP53突变的复发/难治（R/R）CLL患者，经过免疫化疗后复发并发生RT，给予CAR-T细胞联合PD-1单抗治疗，疾病获得完全缓解（CR）。现报道如下并复习相关文献。

## 病例资料

例1，男，70岁，2008年因“反复头晕、乏力”起病，当地医院血常规：WBC 23.5×10^9^/L，淋巴细胞计数（LYM）15×10^9^/L，HGB 151 g/L，PLT 182×10^9^/L。骨髓细胞形态学：成熟淋巴细胞增多，淋巴瘤免疫分型：可见CD5^+^成熟克隆性B淋巴细胞，符合B-CLL/小淋巴细胞淋巴瘤（SLL）表型。B超：颈部、腋下及腹股沟淋巴结肿大，脾脏轻度肿大。诊断为CLL（Binet B期，Rai Ⅱ期）。予氟达拉滨单药治疗1个疗程，后予FC（氟达拉滨+环磷酰胺）方案化疗2个疗程，患者血常规恢复正常，多发肿大淋巴结较前缩小，后予干扰素维持治疗。1年后复查外周血淋巴细胞再次升高，伴淋巴结肿大，予FCR（氟达拉滨+环磷酰胺+利妥昔单抗）方案化疗3个疗程，外周血血常规及肿大淋巴结恢复正常，继续予干扰素维持治疗。2019年5月患者再次出现颈部及腹股沟淋巴结较前明显增大且乏力，遂至我院就诊，血常规：WBC 77.46×10^9^/L，LYM 69.09×10^9^/L，HGB 144 g/L，PLT 102×10^9^/L，β_2_-微球蛋白（β_2_-MG）3.8 mg/L。全身增强CT：全身多发淋巴结肿大，脾脏肿大。完善颈部淋巴结穿刺，病理支持诊断为B-CLL/SLL。进一步行骨髓穿刺。骨髓细胞形态：增生活跃低水平，成熟淋巴细胞32％。免疫分型：分析56％的成熟淋巴细胞群体，见36.1％ CD5^+^成熟克隆性B淋巴细胞，符合B-CLL/SLL表型。淋巴瘤FISH（−）。染色体：伴有11q−的复杂核型。IGHV突变：阳性（突变率3.3％）。二代测序：检测到MYD88、TP53、ARID2基因突变。诊断：CLL/SLL伴del（11q）及TP53突变（CLL-IPI 8分，极高危）。2019年7月16日开始予IR方案（伊布替尼420 mg/d联合利妥昔单抗）治疗。2个疗程后患者肿大淋巴结及脾脏明显缩小，LYM降至33.19×10^9^/L。继续IR方案治疗2个疗程，患者颈部左侧淋巴结再次肿大，遂行颈部淋巴结活检，病理示：瘤细胞CD20（+），CD79a（+），Pax-5（+），CD5（+），Bcl-2（95％+），MUM1（少量散在+），Ki-67（80％+），CD3（−），CD2（−），CD10（−），CyclinD1（−），Bcl-6（−），CD23（−），C-myc（约20％+），TDT（−）；支持诊断弥漫大B细胞淋巴瘤（DLBCL）（倾向活化亚型）。2019年11月13日在IR方案基础上联合miniCHOP方案（半量环磷酰胺+长春新碱+脂质体阿霉素+地塞米松）化疗6个疗程，患者颈部淋巴结缩小，骨髓流式细胞术微小残留病（MRD）转阴，此后继续予IR方案维持治疗，期间患者疾病持续部分缓解（PR）状态。2020年11月7日起开始予维奈托克联合IR方案维持治疗，2021年1月18日复查全身增强CT：右侧腋窝淋巴结较前肿大（19.20 mm×16.17 mm），提示患者出现第4次疾病进展。患者于2021年1月21日应用FB方案（氟达拉滨50 mg，第1、2天；苯达莫司汀125 mg，第1、2天）预处理。2021年1月26日至2021年1月28日患者回输CD19 CAR-T细胞，回输总量1×10^7^/kg。回输后第3天给予PD-1单抗200 mg，期间患者体温正常，无特殊不适，监测各项指标均稳定，无明显细胞因子释放综合征（CRS）。在院期间CAR-T细胞拷贝数变化见图1，回输后107 d及227 d CAR-T细胞拷贝数值分别为140及612 拷贝/µg DNA。2021年2月25日复查全身增强CT提示右侧腋窝肿大淋巴结明显缩小（4.85 mm×5.55 mm），后每3个月予1次PD-1单抗200 mg维持治疗。目前CAR-T细胞治疗后15个月，患者病情稳定。

**图1:**
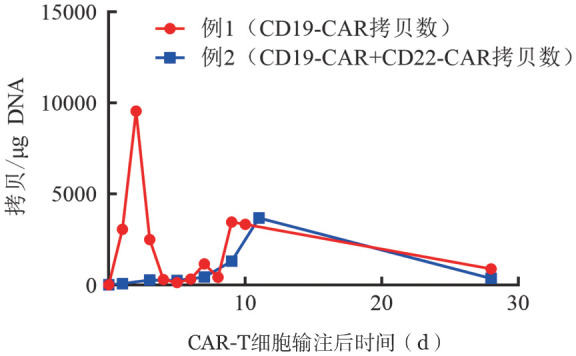
实时荧光定量PCR检测CAR-T细胞治疗后CAR-T细胞拷贝数变化

例2，男，53岁，患者2012年11月体检发现白细胞计数明显增高，骨髓细胞形态学：有核细胞增生活跃低水平，淋巴细胞比例明显增高；淋巴瘤免疫分型：分析76.9％的成熟淋巴细胞群体，CD5、CD19、CD20、CD22、CD23、Kappa阳性，其余阴性，符合B-CLL/SLL表型。B超：全身多发淋巴结肿大。当时诊断为CLL（Binet B期，Rai Ⅰ期）。未予特殊治疗。2019年1月患者乏力伴明显消瘦，予苯丁酸氮芥口服，2019年3月，患者出现反复发热伴明显盗汗，B超示全身多发淋巴结肿大伴肝脾肿大。当地予IR方案治疗。后患者病情稳定。2021年11月患者自觉腹胀加重，至我院就诊，血常规：WBC 25.39×10^9^/L，LYM 21.28×10^9^/L，HGB 123 g/L，PLT 190×10^9^/L。PET-CT示全身多发糖代谢增高淋巴结（SUVmax 5.6，较大者直径3.8 cm），肝脾大，全身骨髓、双侧睾丸（SUVmax 6.0）、脾脏糖代谢增高。行B超引导下腹膜后淋巴结穿刺活检，病理：瘤细胞CD20（+），CD79a（+），CD2（−），CD3（−），Ki-67（约70％+），Bcl-2（约90％+），Bcl-6（约70％+），CD10（−），CyclinD1（−），TdT（−），CD5（−），CD21（−），CD23（−），MUM1（+），c-Myc（−），考虑为弥漫大B细胞型（活化亚型）。完善骨髓穿刺，骨髓细胞形态：淋巴细胞比例增高（成熟淋巴细胞87％）。淋巴瘤免疫分型：分析59.8％的成熟淋巴细胞群体，见55.6％ CD5^+^成熟克隆性B淋巴细胞，符合B-CLL/SLL表型；CLL-FISH：P53缺失80％。IGHV突变阴性。诊断为TP53突变CLL伴RT。2021年12月予ZR-CHOP方案化疗（泽布替尼+利妥昔单抗+环磷酰胺+长春新碱+脂质体阿霉素+地塞米松）。化疗后复查B超示腹腔淋巴结肿大（11.4 cm×6.8 cm）。2022年1月予PD-1单抗+利妥昔单抗+维奈克拉联合治疗2个疗程，腹部CT腹腔淋巴结较前缩小（4.2 cm×2.8 cm），评估病情PR。于2022年2月予FC方案（氟达拉滨50 mg，第1～3天；环磷酰胺0.5 g，第1～3天）预处理，预处理结束后2 d输注CD19及CD22 CAR-T细胞，各靶标总量共1×10^7^/kg，回输后3 d予PD-1单抗200 mg。CAR-T细胞输注后第3～7天体温升高，根据细胞因子等结果评估为CRS 1级，回输后1个月及3个月复查PET-CT均提示腹腔淋巴结明显缩小（1.5 cm×1.2 cm），且无明显葡萄糖代谢升高（图2），提示疾病缓解，目前CAR-T细胞治疗后3个月。

**图2:**
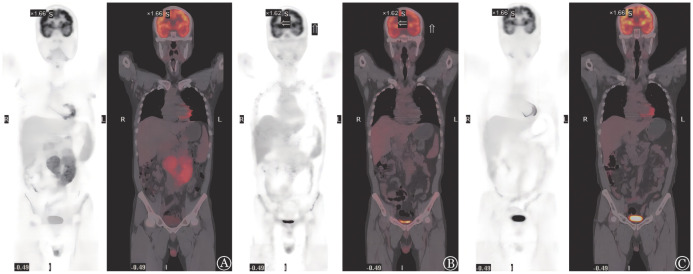
例2治疗前后PET-CT结果比较 A ZR-CHOP方案治疗前；B CAR-T细胞治疗后1个月；C CAR-T细胞治疗后3个月

## 讨论及文献复习

在当前新药时代，CLL患者用药后可能出现克隆演变、旁路活化、微环境变化导致耐药。其中最常见的克隆演变为BTK抑制剂和Bcl-2抑制剂的获得性耐药，约占CLL获得性耐药的80％。本报道中2例患者均伴TP53突变及RT，总体预后极差，虽积极采用含CD20单抗、BTK抑制剂及Bcl-2抑制剂的免疫化疗方案，仍发生耐药且疾病持续进展。近年来CAR-T细胞治疗在B细胞肿瘤中显示出明显效果。Melenhorst等[1]的报道显示，其中心首项CAR-T细胞疗法已经使至少2例侵袭性CLL患者的CR期达到10年。值得注意的是，CAR-T细胞治疗虽可使CLL获得持久缓解，但各研究报道总体反应率差异较大，其中CR率为0～60％[2]–[4]。急性B淋巴细胞白血病患者接受CAR-T细胞治疗的缓解率为68％～93％[5]–[6]，B细胞淋巴瘤患者接受CAR-T细胞治疗的缓解率为64％～86％[7]–[9]，CLL的疗效远低于预期。发生该结果的原因包括：CLL发病机制的重要驱动特征之一是早期免疫缺陷，能促进肿瘤扩张和逃避免疫监视[10]。而化疗进一步加重了T细胞功能缺陷，从而损害CAR-T细胞的活性，该肿瘤微环境与急性淋巴细胞白血病不同，从而导致CAR-T细胞疗效的差异。此外，有相当一部分患者通过抗原阴性逃逸机制导致疾病复发[11]。

多项研究对如何提高CAR-T细胞治疗R/R CLL的疗效进行了探索。首先，临床前研究证实CD19 CAR-T细胞和伊布替尼具有协同作用[12]，患者暴露于至少6个月的伊布替尼可改善CAR-T细胞产品的功能，使肿瘤对CAR-T细胞杀伤敏感，并影响免疫微环境。临床研究也发现，复发/难治性CLL患者应用CD19 CAR-T细胞联合伊布替尼治疗可促进CAR-T细胞的植入和扩增，同时提高缓解率和MRD阴性率，降低3～4级CRS的发生率[13]。其次，Funk等[14]发现PI3K抑制剂可以促进CAR-T细胞线粒体的融合，调控线粒体融合和表观调控，从而增强其体内扩增，增强其抗肿瘤活性。另外，研究发现，CLL来源T细胞表达的免疫检查点分子PD-1发生异常，与健康人群相比，CLL进展期患者有更多的CD4^+^和CD8^+^细胞表达PD-1[15]。同时研究发现B细胞恶性肿瘤患者输注CAR-T细胞后，由于CAR-T细胞表面的PD-1表达增加，导致CAR-T细胞功能下调[16]。上述结果表明，可通过CAR-T细胞结合免疫检查点抑制剂刺激T细胞识别肿瘤细胞。Rupp等[16]以慢病毒为载体运送CRISPR/Cas9编码基因，构建出缺乏PD-1的CAR-T细胞，该方式增强了CAR-T细胞介导的体外肿瘤细胞杀伤及体内PD-L1阳性肿瘤的清除效应。另外一项小样本Ⅰ/Ⅱ期临床试验对CAR-T细胞治疗后出现疾病进展的B-NHL患者应用PD-1单抗联合治疗，结果显示75％（9/12）的患者在第1次给药后外周血CAR-T细胞出现再扩增峰值[17]。此外多项病例报道显示，B-NHL患者采用CAR-T细胞联合PD-1单抗治疗可诱导肿瘤消退，获得长期缓解[18]–[19]。本报道2例患者在使用PD-1单抗后CAR-T细胞拷贝数再次扩增，细胞因子水平再次出现升高，提示T细胞功能进一步活化，例1在后续PD-1单抗维持治疗下监测到CAR-T细胞持续扩增，疾病持续缓解，提示在PD-1单抗的协同作用下CAR-T细胞发挥持久的肿瘤杀伤特性。

目前，使用CAR-T细胞结合免疫检查点抑制剂治疗的临床经验仍处于早期阶段，体内数据多是小样本的报道。现阶段安全性仍是首要考量因素，鉴于两者均有一定程度不良反应，PD-1单抗的使用时机需要谨慎选择。一项小规模前瞻性研究在输注CAR-T细胞后第3天加用PD-1单抗，显示加用PD-1单抗治疗后促进CAR-T细胞再激活，但并未观察到显著CAR-T细胞再激活引起的CRS、中枢神经系统不良反应及CAR-T细胞相关不良反应[20]。一项CD30 CAR-T细胞输注后两周起固定疗程应用PD-1单抗的临床研究显示，仅25％（1/4）的患者出现CRS，总体反应率达100％[21]。本报道两例患者均于回输后早期联合应用PD-1单抗，虽然均再次出现CAR-T细胞扩增，但CRS未增加。因此，以上结果均显示无论PD-1单抗抢先治疗还是维持治疗，CAR-T细胞联合PD-1单抗治疗安全可控。需要注意的是，一项回顾性研究纳入39例R/R淋巴瘤患者，应用PD-1单抗后行异基因造血干细胞移植，应用PD-1抑制剂后患者致死性Ⅳ级急性移植物抗宿主病发生率高，且疗效差[22]。因此，对于异基因造血干细胞移植患者，特别是需要桥接移植的患者或移植后患者，PD-1单抗的选择需尤其慎重。另外，值得一提的是，本报道例1因年龄较大且首次采用联合免疫治疗，预处理方案选择FB方案。既往研究显示，该方案疗效与FC方案相似，但可降低CRS、免疫效应细胞相关神经毒性综合征、感染及血液学不良反应[23]。该患者无明显CRS反应，证实该方案可降低CAR-T细胞相关不良反应，但后期仍需扩大样本设计前瞻性试验进一步研究。

综上，本中心报道了2例伴TP53突变的R/R CLL患者，采用BTK抑制剂联合利妥昔单抗治疗后发生DLBCL-RT，予免疫化疗后疾病再次进展，遂予CAR-T细胞联合PD-1单抗治疗，两例患者均获得CR，且未出现显著CRS相关不良反应。CAR-T细胞联合PD-1单抗方案为老年、高危、难以接受移植的患者提供了新的治疗策略，而对于年轻、遗传学高危患者后续是否需要桥接异基因造血干细胞移植、何时桥接移植，有待进一步探索。
